# Nonrepresentativeness of Human Mobility Data and its Impact on Modeling Dynamics of the COVID-19 Pandemic: Systematic Evaluation

**DOI:** 10.2196/55013

**Published:** 2024-06-28

**Authors:** Chuchu Liu, Petter Holme, Sune Lehmann, Wenchuan Yang, Xin Lu

**Affiliations:** 1 School of Economics and Management Changsha University of Science and Technology Changsha China; 2 College of Systems Engineering National University of Defense Technology Changsha China; 3 Department of Computer Science Aalto University Espoo Finland; 4 Center for Computational Social Science Kobe University Kobe Japan; 5 Department of Applied Mathematics and Computer Science Technical University of Denmark Copenhagen Denmark

**Keywords:** human mobility, data representativeness, population composition, COVID-19, epidemiological modeling

## Abstract

**Background:**

In recent years, a range of novel smartphone-derived data streams about human mobility have become available on a near–real-time basis. These data have been used, for example, to perform traffic forecasting and epidemic modeling. During the COVID-19 pandemic in particular, human travel behavior has been considered a key component of epidemiological modeling to provide more reliable estimates about the volumes of the pandemic’s importation and transmission routes, or to identify hot spots. However, nearly universally in the literature, the representativeness of these data, how they relate to the underlying real-world human mobility, has been overlooked. This disconnect between data and reality is especially relevant in the case of socially disadvantaged minorities.

**Objective:**

The objective of this study is to illustrate the nonrepresentativeness of data on human mobility and the impact of this nonrepresentativeness on modeling dynamics of the epidemic. This study systematically evaluates how real-world travel flows differ from census-based estimations, especially in the case of socially disadvantaged minorities, such as older adults and women, and further measures biases introduced by this difference in epidemiological studies.

**Methods:**

To understand the demographic composition of population movements, a nationwide mobility data set from 318 million mobile phone users in China from January 1 to February 29, 2020, was curated. Specifically, we quantified the disparity in the population composition between actual migrations and resident composition according to census data, and shows how this nonrepresentativeness impacts epidemiological modeling by constructing an age-structured SEIR (Susceptible-Exposed-Infected- Recovered) model of COVID-19 transmission.

**Results:**

We found a significant difference in the demographic composition between those who travel and the overall population. In the population flows, 59% (n=20,067,526) of travelers are young and 36% (n=12,210,565) of them are middle-aged (*P*<.001), which is completely different from the overall adult population composition of China (where 36% of individuals are young and 40% of them are middle-aged). This difference would introduce a striking bias in epidemiological studies: the estimation of maximum daily infections differs nearly 3 times, and the peak time has a large gap of 46 days.

**Conclusions:**

The difference between actual migrations and resident composition strongly impacts outcomes of epidemiological forecasts, which typically assume that flows represent underlying demographics. Our findings imply that it is necessary to measure and quantify the inherent biases related to nonrepresentativeness for accurate epidemiological surveillance and forecasting.

## Introduction

With large-scale empirical data (eg, mobile phone records, GPS data, and location-based social network data) becoming available with increasingly fine spatial and temporal resolution [1], quantitative studies on individual and collective mobility patterns have flourished in the past few years [2-6]. These developments have offered advances with respect to understanding migratory flows, traffic forecasting, urban planning, and epidemic modeling [7-10]. The ongoing COVID-19 pandemic has further intensified discussions on how to optimally use human mobility research to support outbreak responses and nonpharmaceutical interventions (eg, contact tracing) [11-14].

The representativeness of data sets used to infer real-world human mobility, however, has typically not been explicitly incorporated in such analyses. This is potentially troubling as representativeness is known to be especially poor for socially disadvantaged minorities such as low-income groups, women, children, and older people. For example, it has been confirmed that individuals’ probability of travel is not randomly or equally distributed, and there is significant heterogeneity when comparing the travel patterns of different demographic groups [15-17]. For example, women are more localized than men in their movements and visit fewer locations in regions such as Latin America, Bangladesh, and sub-Saharan Africa [18,19]. In the specific case of epidemic outbreaks, low-income individuals are not necessarily able to limit their exposure to a circulating virus by reducing mobility and must continue, for example, commuting behavior to remain employed. Thus, this group is subject to a substantially higher probability of becoming infected in an epidemic than higher-income groups [20]. Further, different contact rates across age groups have been observed in COVID-19 incidence cases [21], and higher COVID-19 infection rates among disadvantaged racial and socioeconomic groups have been observed in multiple studies [22,23]. It has also been argued that including information about demographic heterogeneity in human mobility patterns, for example, by combining demographically stratified travel data with epidemiology research, would make epidemiological models more robust [24].

While it is widely recognized that rich new data sources can provide near–real-time information about human mobility [25] and powerful input into models that estimate imported cases using regional mobility information when modeling pathogen transmission, the state-of-the-art models do not consider data representativeness. This is typically because for privacy concerns, most data sets are not disaggregated demographically. Instead, relevant information on demographic features and social relationships is traditionally collected by censuses and other surveys [26-28].

As we argue below, however, simply considering the population demographics at the origin of a trip does not represent the traveling population.

To systematically evaluate how real-world travel flows differ from census-based estimations, we use an aggregated and anonymized data set collected from 318 million mobile phones. Specifically, we quantified the disparity in the population composition between actual migrations and resident composition according to census data and found significant differences. We then investigated how this nonrepresentativeness impacts epidemiological modeling. The aim of this study is to illustrate the nonrepresentativeness of data on human mobility and the impact of this nonrepresentativeness on modeling dynamics of the COVID-19 pandemic.

## Methods

### Data Description

In China, a total of 847 million Chinese people use mobile phones to surf the internet, accounting for 99.1% of the total netizens. The penetration rate of mobile phone usage among the population aged 15-65 years is almost 100%, providing extensive coverage and high representativeness for the national population. To understand the demographic composition of population movements, we collected nationwide mobility data from 318 million mobile phone users in China from January 1 to February 29, 2020. All population flow data were aggregated on the basis of users’ geographic locations and demographic characteristics (eg, gender and age). To enhance extrapolation and representation of the population, a machine learning method was used to extrapolate the data to all users of the entire network, which also agrees well with the official population statistics (*R^2^*=0.98; Multimedia Appendix 1 [2,29-31]).

### Epidemiological Modeling

To illustrate the impact of data nonrepresentativeness on modeling dynamics of the COVID-19 epidemic, we constructed an age-structured SEIR (Susceptible-Exposed-Infected- Recovered) model of COVID-19 transmission developed by Prem et al [32]. In fitting this age-mixing transmission model with heterogeneous contact rates between age groups [33], the differential age composition of traveling people and the overall national population were input as alternative parameters. By comparing model outputs, we measured the bias caused by the data nonrepresentativeness of demographic composition in forecasting epidemic dynamics.

### Ethical Considerations

The study data provided by the operator were anonymized (without personally identifying information) and aggregated at the city level. As no individual study was carried out, no ethical approval was required to undertake this scoping study.

## Results

### Demographic Heterogeneity Among Traveling Individuals

For our analysis, we draw on a unique data set from China. China is an ideal location to study representativeness in mobile phone data because of its very high smartphone penetration. In China, the penetration rate of mobile phone usage among the population aged 15-65 years is almost 100%, providing extensive coverage and high representativeness for the national population [29]. We estimate the full national mobility at the city level by extrapolating from 318 million mobile phone users (see Multimedia Appendix 1 [2,29-31] for details).

**Figure 1:**
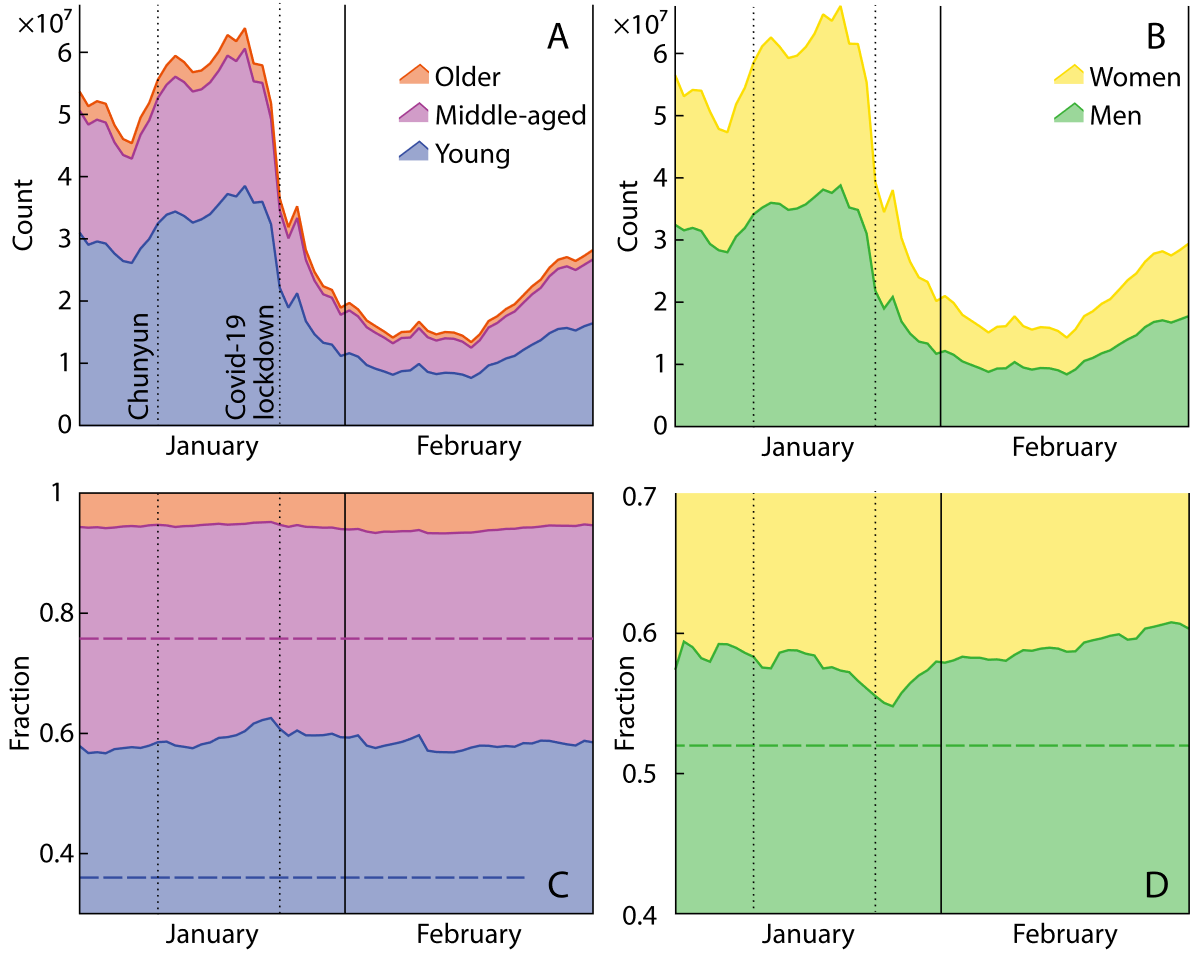
Profiles of the intercity movements extracted from mobile phone data between January 1 and February 29, 2020, in China. (A) and (B) show the daily number of travelers for different age and gender groups; (C) and (D) show the respective ratios. Dashed horizontal lines denote the composition of respective groups in the latest 7th census. As children generally do not have mobile phones, the proportions of young (20-39 years), middle-aged (40-59 years), and older people (≥60 years) add up to 100%.

Our comparison reveals a marked difference between the overall population composition and those who travel. Hereinafter, we define “young” individuals as those in their 20s-30s, “middle-aged” individuals as those in their 40s-50s, and “older adults” as those older than 60 years. Specifically, we found that the majority of population flows within China are generated by men and young people. Although mobility behavior fluctuated strongly across our observation period (Figures 1A and 1B) and is affected by temporal factors such as weekdays and holidays, the composition of travelers did not change significantly over different periods (Figures 1C and 1D). In the population flows, 59% (n=20,067,526) of travelers are young and 36% (n=12,210,565) of them are middle-aged (as children generally do not have mobile phones, all ratios are calculated with minors younger than 18 years having been excluded). This ratio is completely different from what we observed in the overall adult population composition of China (about 1084 million in total) [30], where 36% (n=390 million) of individuals are young and 40% (n=430 million) of them are middle-aged. Furthermore, daily male travelers constitute approximately 59% (n=20,858,026) of the total number of traveling individuals, which is greater than the overall proportion of men (51.2%, n=721 million). Compared to men, women travel less often, but we found that when women travel, they tend to move slightly further than men, with 175 km traveled per person in an average intercity trip, compared to 170 km for men (*P*<.001).

### Bias From Data Nonrepresentativeness

Since individual mobility is the primary reason for the spatial diffusion of an epidemic, it is important to directly explore how the demographic heterogeneity of human migration behaviors impacts our ability to forecast the spatial behavior of epidemics.

**Figure 2:**
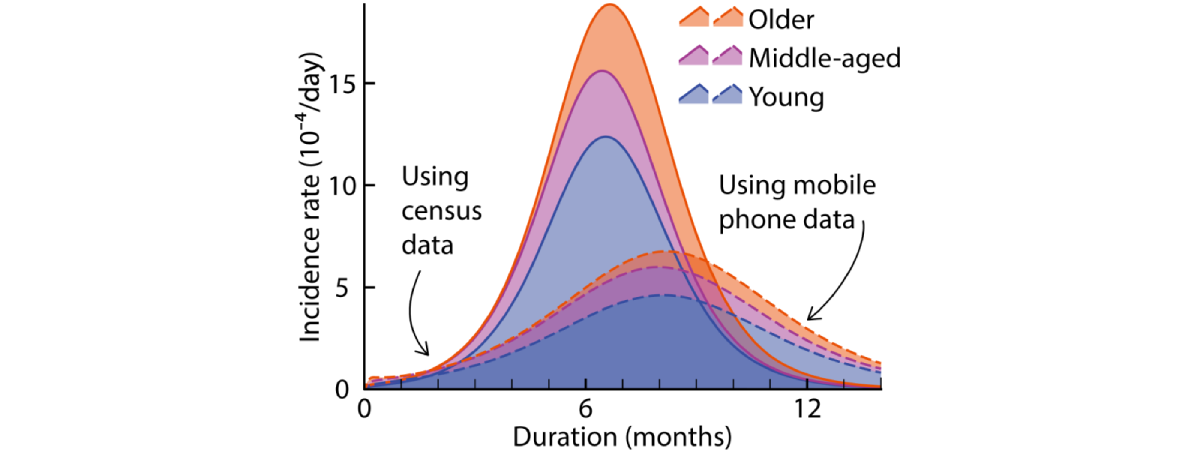
Dynamics of the incidence rates among different groups predicted by the age-structured model. Solid lines indicate the incidence rates of different age groups from census data, and dashed lines indicate the incidence rates by using traveling data from mobile phones.

By fitting an age-structured transmission model [32,33], and including differential age composition as an input parameter, we measured the possible bias caused by the nonrepresentativeness of data on the traveling population in modeling epidemic dynamics. Feeding the composition of travelers and composition from the census data separately into the model, we found that the predicted number of infected individuals had a striking bias: the maximum number of daily infections in these 2 populations differ by nearly 3 times (521 infected individuals among a total of 1 million people for the composition of travelers and 1521 infected individuals for the census population), and their peak time had a large gap of 46 days. Although older adults are the most susceptible population, the 2 infection rates among older people collected from mobility data and census data deviate strongly (Figure 2). Further, while the predicted cumulative number of confirmed cases do gradually stabilize late in the epidemic, the gap between the results of the 2 models is nonnegligible (with a deviation of around 79.5%) with respect to infection volumes. Failing to include information about age and gender structure in real-world human mobility is thus likely to introduce considerable biases in epidemiological studies, especially in the early phase of an epidemic outbreak caused by imported cases [24].

## Discussion

By comparing mobility traces from mobile phone users to census data, our study has highlighted a number of striking differences in the demographic composition of those who travel with respect to the overall population. For example, we found that 59% (n=20,067,526) of travelers are young and 36% (n=12,210,565) of them are middle-aged, which is completely different from the composition of the adult population in China, where 36% of people are young and 40% of them are middle-aged. The travel probability and travel distance between men and women were also significantly different. This realization is especially important in the case of epidemic forecasting, and increased awareness of this issue in the scientific community has the potential to improve not only epidemiological models but also our overall understanding of possible biases when inferring human mobility from cell phone data and the representational issues of mobility data. It is important to emphasize that while China is an ideal place to study representativeness, our findings about which fraction of individuals compose the population of travelers are specific to China. The fraction of young, middle-aged, old, male, and female individuals who travel is likely to depend on a range of factors and can be expected to be different in different countries.

Nonetheless, the realization that understanding the representativeness of mobility data is crucial for epidemic monitoring and forecasting is generalizable. Thus, our results imply that when generalizing results from population mobility analysis, these differences should be included in the analysis to avoid potential biases caused by data nonrepresentativeness. For example, in the case of the COVID-19 pandemic, as travelers often have a higher probability of infection, the transmission risk among men and youth could be a promising focus for COVID-19 prevention.

In the recent Omicron waves, imported infections represented the majority of cases in China, and most COVID-19–positive individuals had a travel history to high-risk areas such as Shanghai [34]. As presymptomatic and asymptomatic pathogen carriers can travel to a foreign country and initiate the spread of COVID-19 even when there is no community transmission, human migration behaviors are promising candidates to incorporate into epidemiological models. Our findings emphasize that focusing on the representativeness of mobility data is essential for more sophisticated modeling approaches to capture key mechanisms of epidemic propagation. In future work, we intend to further explore how to accurately quantify the inherent biases related to data nonrepresentativeness for accurate epidemiological surveillance and forecasting.

## Appendix

Multimedia Appendix 1 Underrepresented data of the population flow.

## Abbreviations

SEIR: Susceptible-Exposed-Infected-Recovered

## Data Availability

Deidentified data and code used in the analysis are available upon reasonable request from the corresponding author.
