# Supplementary material for: Nonrepresentativeness of Human Mobility Data and its Impact on Modeling Dynamics of the COVID-19 Pandemic: Systematic Evaluation
Source: JMIR Form Res. 2024 Jun 28;8:e55013. doi: 10.2196/55013 (PMC11245661; doi:10.2196/55013)
Supplement: Multimedia Appendix 1 [file formative_v8i1e55013_app1.docx]

Multimedia Appendix 1

Materials and Methods

Table S1. The description of demographic results and independent variables in the epidemic model.

Fig. S1. Consistency of mobile phone users’ estimates with the population data for central prefectures at the district level.

Fig. S2. The daily inflow number and outflow number of each province in mainland China.

Fig. S3. The daily inflow number and outflow number of people in different age groups in each province of mainland China.

Fig. S4. The age composition of inflow and outflow migrants in each province of mainland China.

Fig. S5. The daily inflow number and outflow number of men and women in each province of mainland China.

Fig. S6. The gender composition of inflow and outflow migrants in each province of mainland China.

Fig. S7. The per capita travel distance of different populations.

Fig. S8. Daily travel distance per capita of people in different age groups.

**Materials and Methods**

Data Description

To understand the demographical composition of population movements, we collected nationwide mobility data from 318 million mobile phone users in China from Jan. 1, 2020 to Feb. 29, 2020. The period ranges from the Chinese New Year travel season (chunyun), to the exceptional calm of COVID-19 lockdowns, and then to the recovery periods.

The mobile phone data used for this study were aggregated from cellular signaling data (CSD), which are collected by China Unicom (one of the largest national mobile carriers in China) with rich spatio-temporal information. The data contains real-time base-station-dependent location information from mobile phones, which is recorded when users are making phone calls, sending messages, or switching on/off their devices. As the CSD is recorded as long as there is an active or passive positioning data event, the temporal resolution is at the minimum of every 30 minutes per record. To exclude a large number of users who only briefly pass through a prefecture, users who stay in a prefecture for less than half an hour were filtered out.

All population flow data has been aggregated based on users' geographic locations and demographic characteristics (e.g., gender and age). For the purpose of emergency response, all mobility data provided by the operator were anonymized (without personally identifying information) and aggregated. The authors only had access to exported and aggregated data at the city level.

Data Extrapolation and Validation

To enhance the extrapolation and representation of the population, the operator helped to extrapolate the data to all users of the entire network. User coverage, call ratio with other operators, and various parameters extracted from users’ age, gender, etc., were combined and modeled by using a machine learning method.

A comprehensive validation analysis on both user coverage and data representativeness was implemented by Tan et al (2021)^^[[1]](#footnote-1)^^. They verified that the extrapolation results from the operator were in good agreement with GPS-based LBS (location-based services) data and official estimates on population flow during the study period. And it was proven that there is a high consistency between mobile phone data estimates and population flows generated by Baidu LBS (location-based services)^^[[2]](#footnote-2)^^, one of the most widely used open sources (Pearson correlation, *r=0.96*, *p<0.01*). In addition, the consistency between the population estimated from mobile phone data and the official statistics on the residential population for central prefectures at the district level (Fig. S1) was evaluated (*R^2^=0.98*).


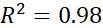


Epidemical Modeling

To illustrate the impact of data non-representativeness on modeling epidemic dynamics, the age-structured SEIR (Susceptible-Exposed-Infected-Recovered) model for the Wuhan COVID-19 outbreak proposed by Prem et al.^^[[3]](#footnote-3)^^ was reconstructed. Due to the differences in physical health and vaccination status of people of different ages, the infection probability in COVID-19 is generally considered to be disparate in differential age groups. In the SEIR model proposed by Prem et al., an age-specific susceptibility to infection of individuals was also set. The heterogeneous contact rates between age groups used in our simulation were surveyed by Zhang et al.^^[[4]](#footnote-4)^^. We kept the contact rate of each population unchanged and aggregated the rate of each age group as a parameter of our simulation model. Both the age composition of traveling people and the overall national population in China’s Census data were input to the model as alternative parameters. By comparing the two model outputs, we measured the bias caused by the data non-representativeness of demographic composition in forecasting epidemic dynamics.

Provincial Comparison

For each province, daily inflows are basically equal to daily outflows, but there are two notable mobility patterns during the chunyun migration period that occurs over the Lunar New Year holiday. In the most developed regions of China (i.e., the labor-input areas), such as Beijing, Shanghai and Zhejiang province, the outflow population is significantly more than the inflow population. By contrast, in labor-export areas, such as Shanxi province and Guangxi province, the number of inflow people is notably higher than the number of outflows (Fig. S2).

In addition, the age composition of migrant workers also varies across provinces (Fig. S3). Specifically, laborers from economically underdeveloped regions (e.g., Yunnan province, Guizhou province, and Gansu province) mainly consist of young people in their 20s. But in the northeastern provinces (i.e., Jilin, Liaoning, Heilongjiang), whose average wages are reported to be the lowest in China, middle-aged migrant laborers still account for considerable proportions (over 40%) (Fig. S4).

Travel Distance

We found that there is a negative correlation between the city distance and the number of mobility population (Pearson correlation coefficient, *r=-0.67*), but as the distance increases, this negative correlation surprisingly weakens. Young people in their 20s always have the longest travel distance, and the older the population, the shorter their average mobility distance (Fig. S8) (Minors below 18 years old are excluded, as children in China generally do not have mobile phones). Besides, the travel distance of female mobility was observed to be farther than the distance of male travel (Fig. S7), indicating that men account for the greater proportion of short-distance movements in China.

Table S1. The description of demographic results and independent variables in the epidemic model.

| **Demographic groups in traveling people (relative ratio)** | | | | | | |
| --- | --- | --- | --- | --- | --- | --- |
| Age composition $A_{t}$ | youth | | | middle-aged | | elder |
|  | 59% | | | 36% | | 5% |
| Gender composition $G_{t}$ | male | | | female | |  |
|  | 59% | | | 41% | |  |
| **Demographic groups in overall population (relative ratio, from Census)** | | | | | | |
| Age composition $A_{c}$ | youth | | | middle-aged | | elder |
|  | 36% | | | 40% | | 24% |
| Gender composition $G_{c}$ | male | | | female | |  |
|  | 51% | | | 49% | |  |
| **Parameters in epidemical modeling** | | | | | | |
| **Age composition** | | | | | | |
| $A_{c}$  Age composition from Census data | | | $A_{t}$  Age composition of traveling people | | | |
| **Age-structured contact rate** $\boldsymbol{r}$ | | | | | | |
| $r_{(youth, youth)}$ | | $r_{(youth, middle\_aged)}$ | | | $r_{(youth, elder)}$ | |
| $r_{(middle\_aged, middle\_aged)}$ | | $r_{(middle\_aged, elder)}$ | | | $r_{(elder, elder)}$ | |


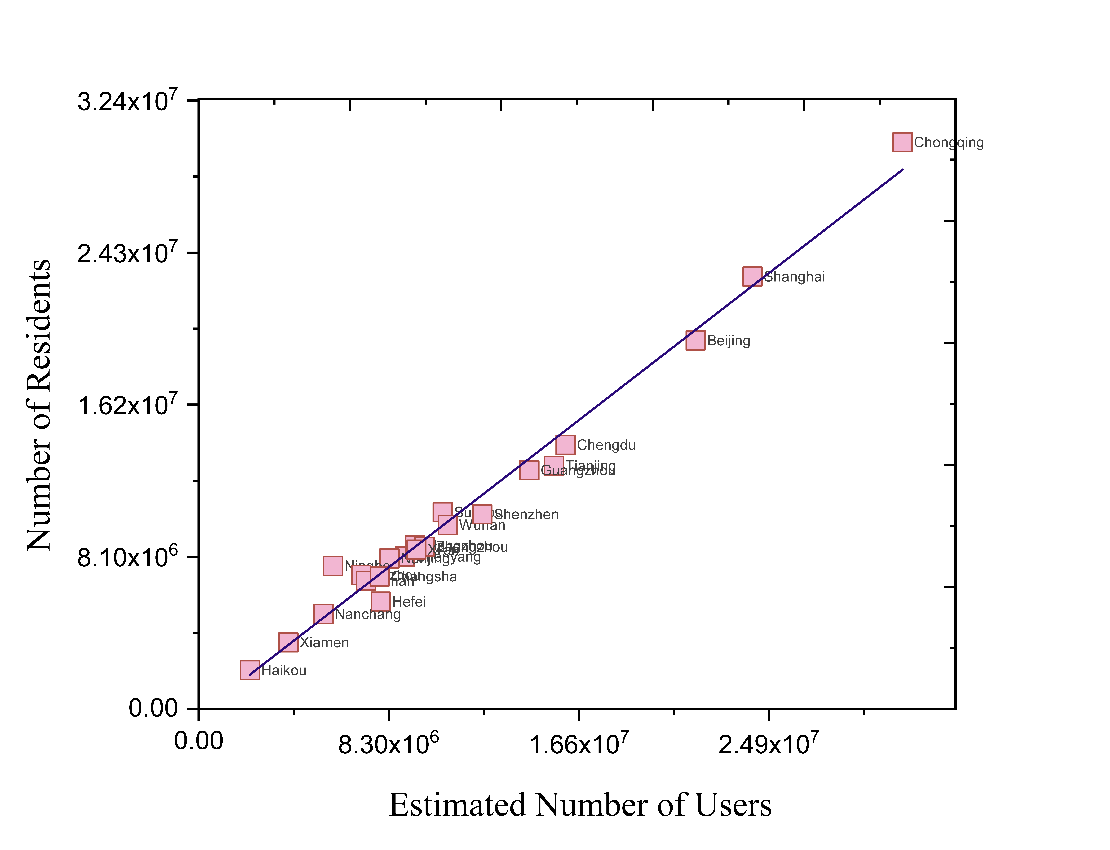


Fig. S1. Consistency of mobile phone users’ estimates with the population data for central prefectures at the district level, adjusted *R^2^=0.98*.


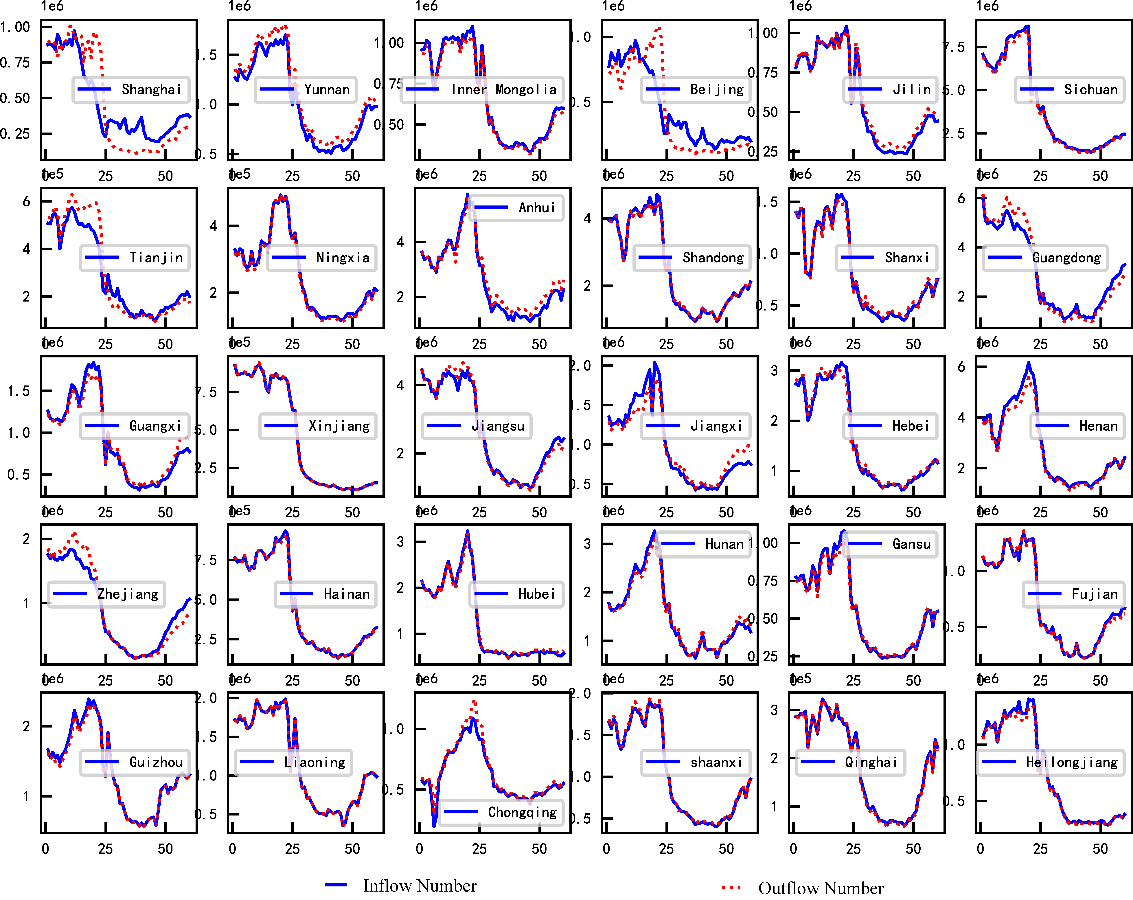


Fig. S2. The daily inflow number and outflow number of each province in mainland China. The horizontal axis represents date (for clear visualization, date 1 represents Jan. 1, 2020, so that date 60 is Feb. 29, 2020); the vertical axis shows the number of migrants. The solid line describes daily inflow number, and the dashed line describes daily outflow number.


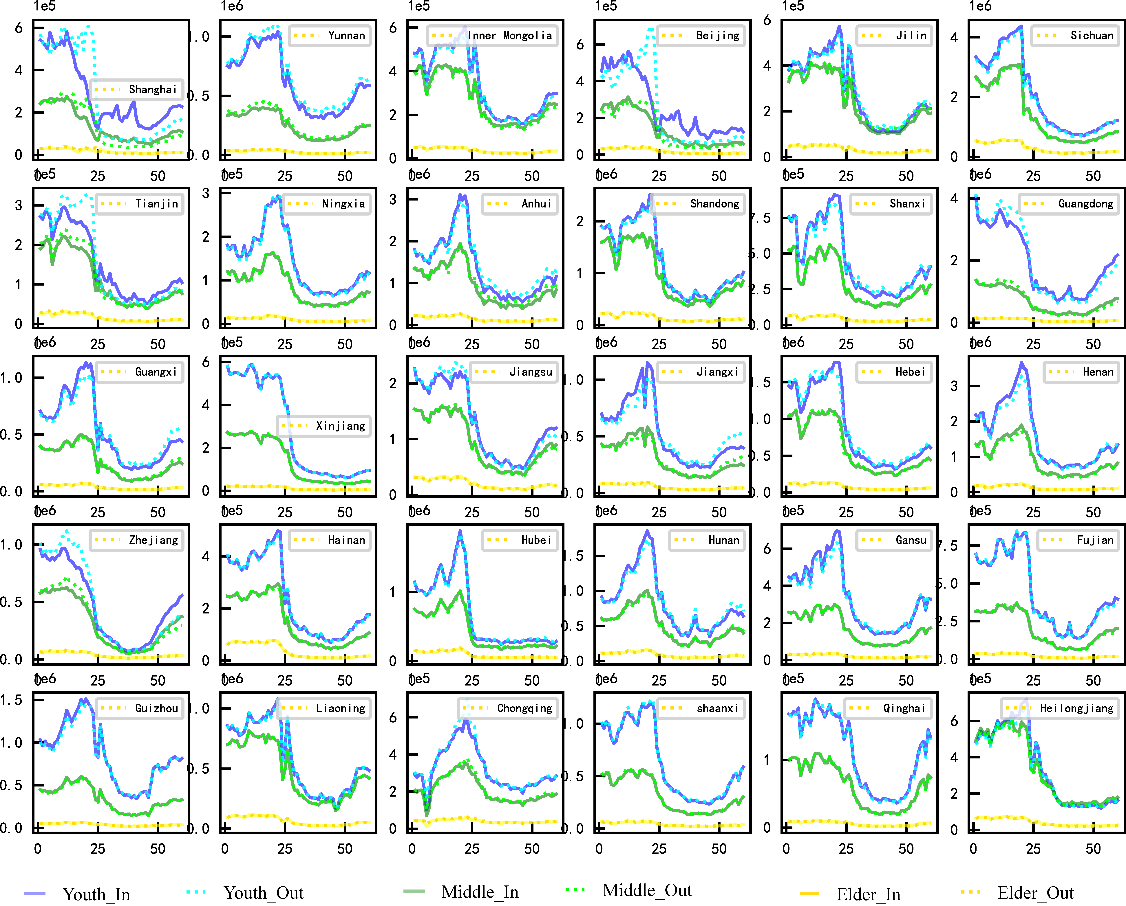


Fig. S3. The daily inflow number and outflow number of people in different age groups in each province of mainland China. The horizontal axis represents date; the vertical axis shows the number of migrants. The solid line describes daily inflow number, and the dashed line describes daily outflow number. The youth are people in their 20s and 30s, the middle-aged groups are people in their 40s and 50s, and the elder are people over 60s.


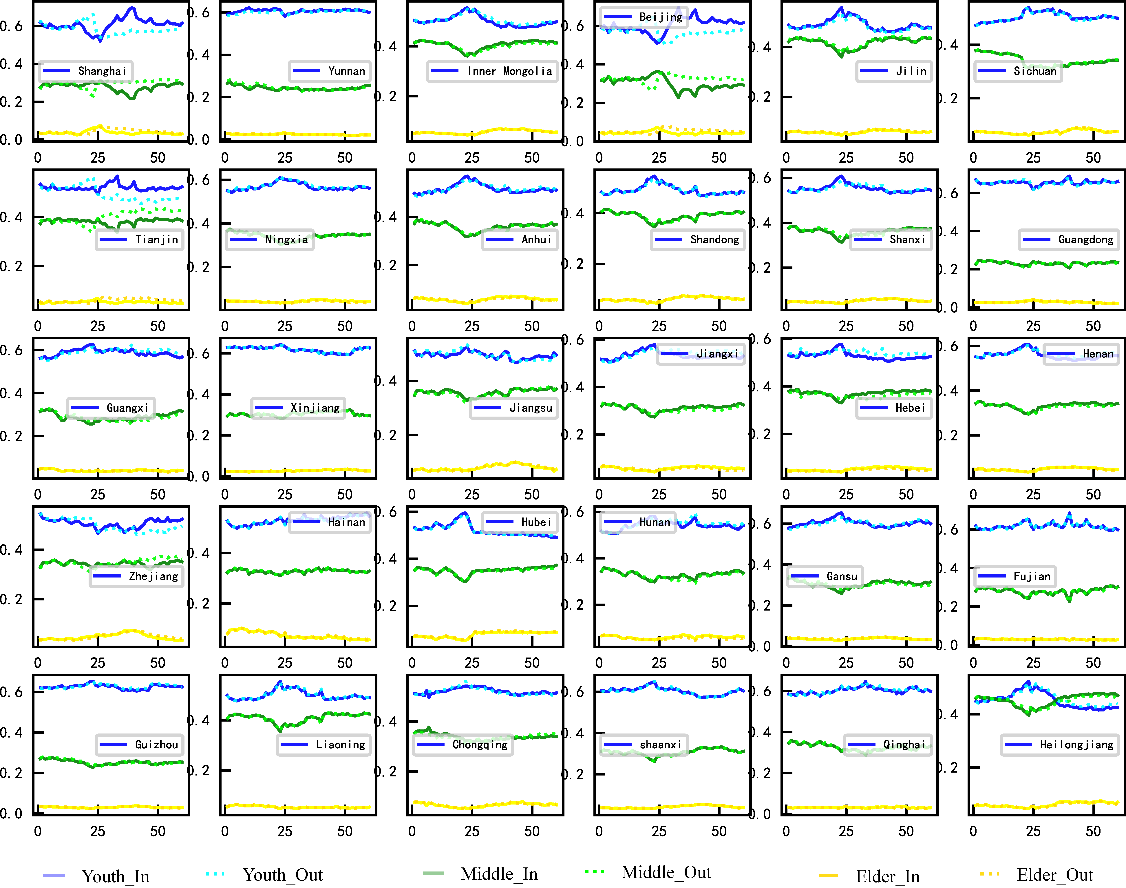


Fig. S4. The age composition of inflow and outflow migrants in each province of mainland China. The horizontal axis represents date; the vertical axis shows the proportion of various populations. The solid line describes daily inflow number, and the dashed line describes daily outflow number. The youth are people in their 20s and 30s, the middle-aged groups are people in their 40s and 50s, and the elder are people over 60s.


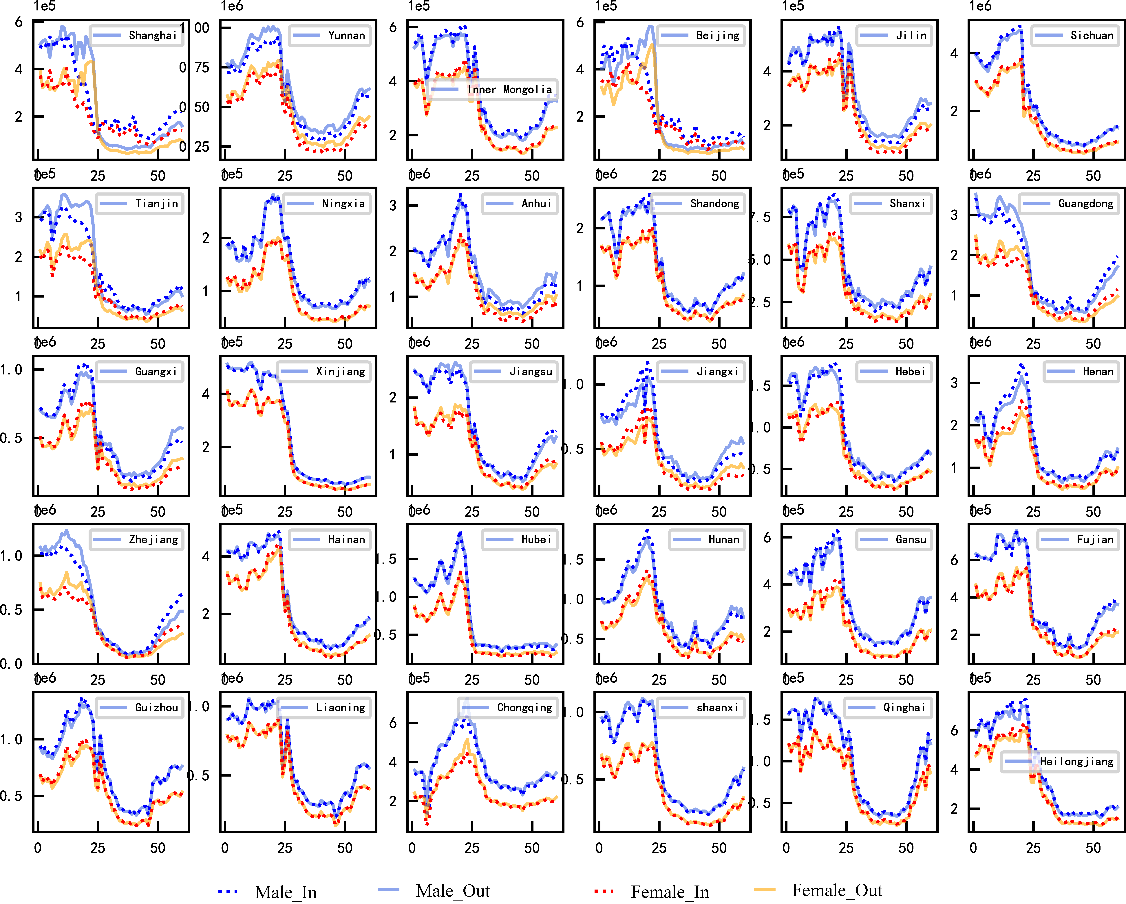


Fig. S5. The daily inflow number and outflow number of men and women in each province of mainland China. The horizontal axis represents date; the vertical axis shows the number of migrants. The solid line describes daily outflow number, and the dashed line describes daily inflow number.


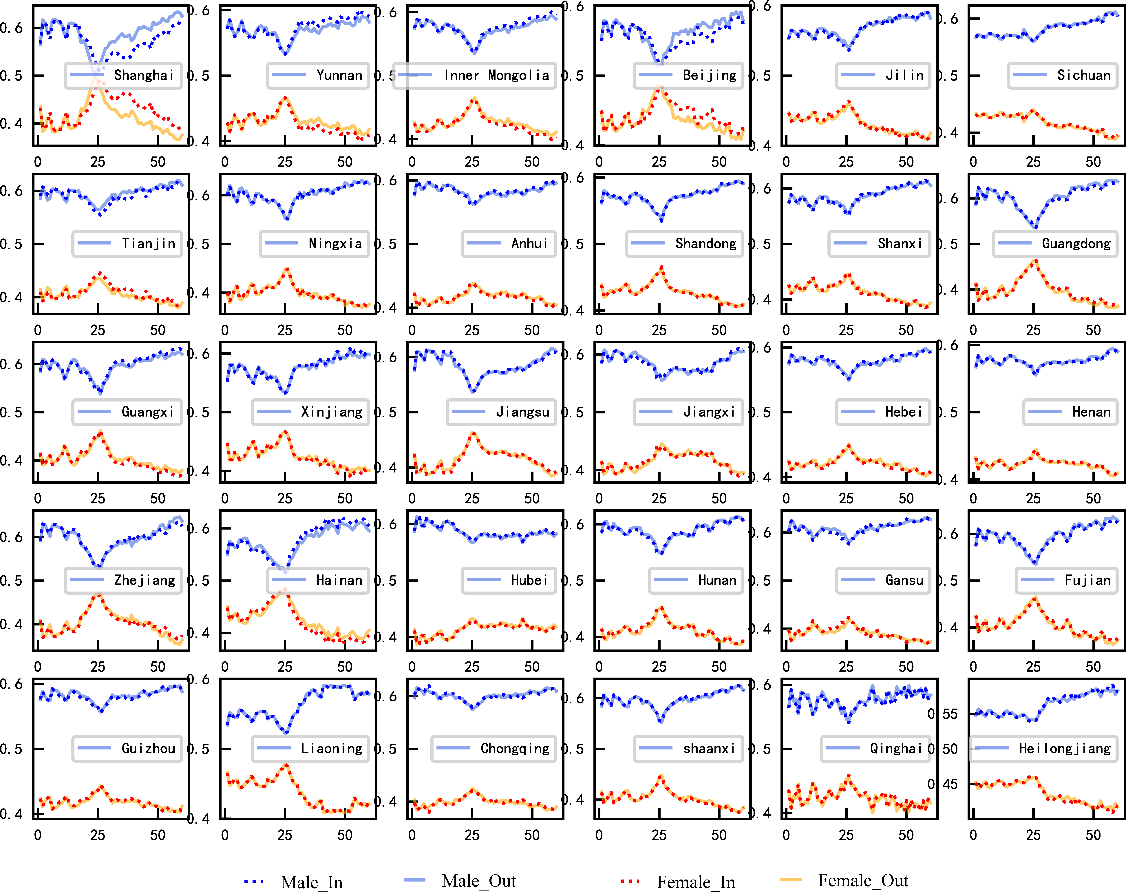


Fig. S6. The gender composition of inflow and outflow migrants in each province of mainland China. The horizontal axis represents date; the vertical axis shows the proportion of males or females. The solid line describes daily outflow number, and the dashed line describes daily inflow number.


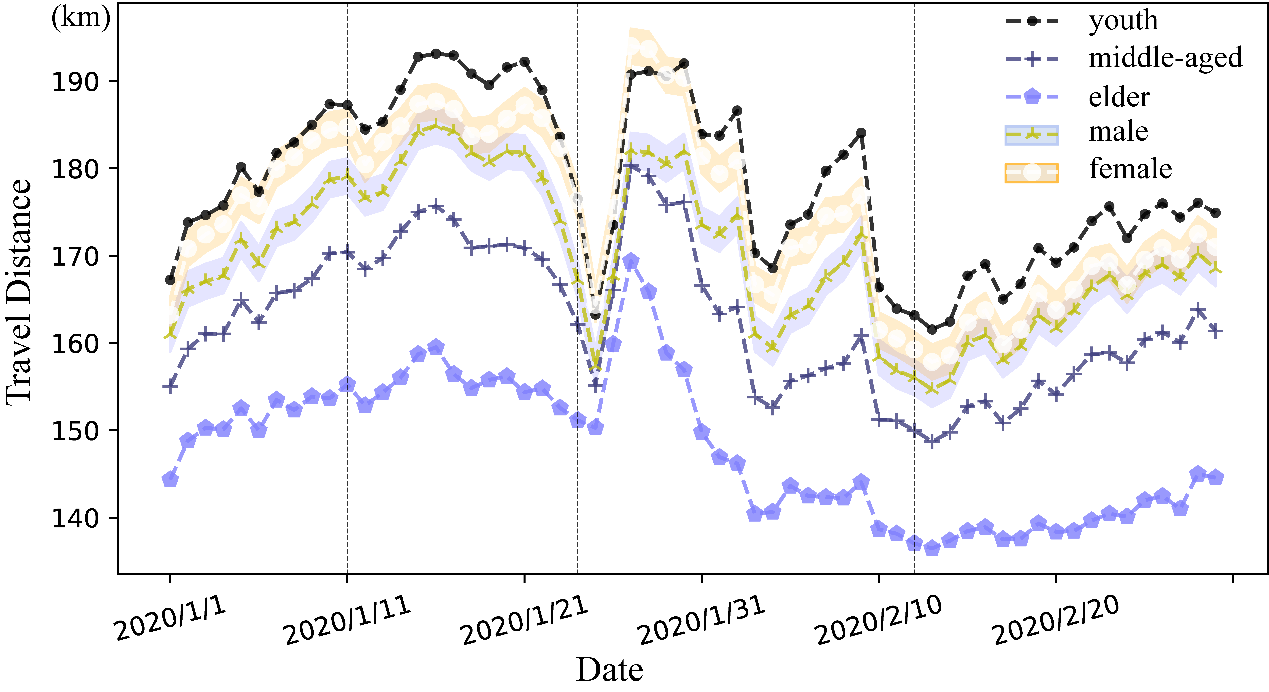


Fig. S7. The per capita travel distance of different populations.


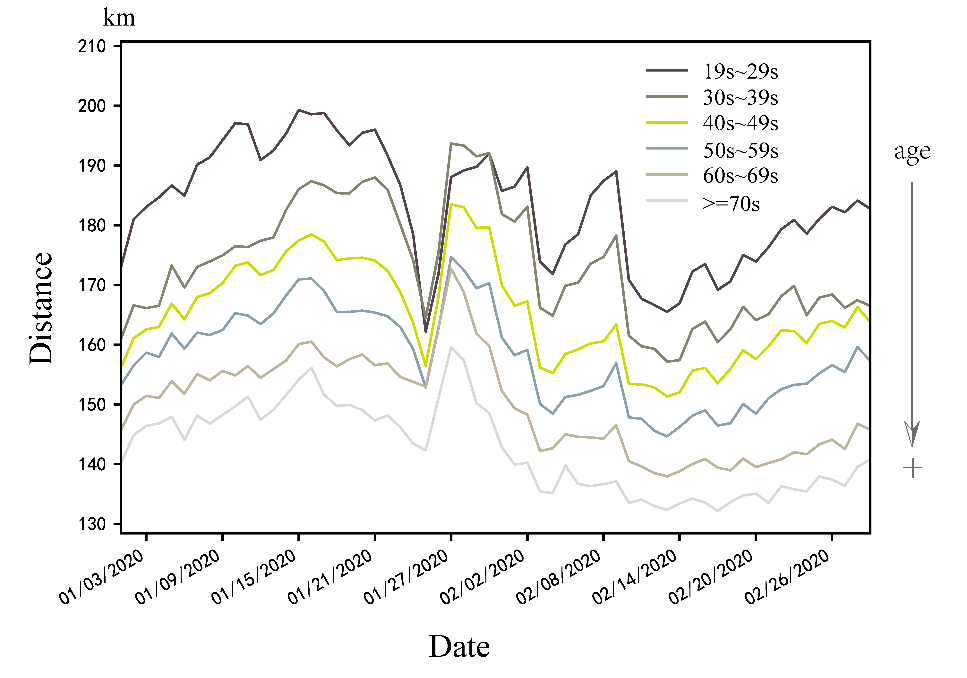


Fig. S8. Daily travel distance per capita of people in different age groups.

1. Tan S, Lai S, Fang F, et al. Mobility in China, 2020: a tale of four phases. National Science Review, 2021, 8(11): nwab148. [↑](#footnote-ref-1)
2. <https://qianxi.baidu.com/>. [↑](#footnote-ref-2)
3. Prem K, Liu Y, Russell T W, et al. The effect of control strategies to reduce social mixing on outcomes of the COVID-19 epidemic in Wuhan, China: a modelling study. The Lancet Public Health, 2020, 5(5): e261-e270. [↑](#footnote-ref-3)
4. Zhang J, Litvinova M, Liang Y, et al. Changes in contact patterns shape the dynamics of the COVID-19 outbreak in China. Science, 2020, 368(6498): 1481-1486. [↑](#footnote-ref-4)
